# Supplementary material for: Individual Nutrition Is Associated with Altered Gut Microbiome Composition for Adults with Food Insecurity
Source: Nutrients. 2022 Aug 19;14(16):3407. doi: 10.3390/nu14163407 (PMC9416073; doi:10.3390/nu14163407)

SUPPLEMENTARY TABLES

Table S1. Covariate Associations. Results from generalized linear models with covariates only.

	MNI[1]		Electrolyte Index[1]		Macronutrient Index[2]		Mineral Index[1]		Vitamin Index[1]	
**Characteristic**	**β (95% CI)**	**p-value**	**OR (95% CI)**	**p-value**	**OR (95% CI)**	**p-value**	**OR (95% CI)**	**p-value**	**OR (95% CI)**	**p-value**
Antibiotic use in the past year										
Did not use	—		—		—		—		—	
Did use	0.01 (-3.5, 3.6)	>0.9	1.08 (0.76, 1.53)	0.7	1.09 (0.76, 1.58)	0.6	0.95 (0.68, 1.34)	0.8	0.78 (0.41, 1.43)	0.4
Unknown/Missing	-3.3 (-11, 4.0)	0.4	0.96 (0.46, 2.00)	>0.9	1.31 (0.59, 2.80)	0.5	0.71 (0.34, 1.43)	0.3	3.25 (1.27, 7.68)	0.009
Poverty:Income Ratio	0.31 (-0.40, 1.0)	0.4	1.05 (0.98, 1.13)	0.2	1.02 (0.95, 1.09)	0.6	0.99 (0.93, 1.07)	0.9	1.02 (0.92, 1.14)	0.7
Education										
<High school	—		—		—		—		—	
High school or associate's degree	7.2 (-0.14, 15)	0.055	3.22 (1.42, 8.28)	0.009	2.28 (0.96, 6.32)	0.081	0.66 (0.32, 1.34)	0.2	1.61 (0.43, 10.5)	0.5
Bachelor's degree or higher	11 (3.4, 19)	0.005	3.48 (1.47, 9.22)	0.007	1.81 (0.73, 5.17)	0.2	0.96 (0.45, 2.04)	>0.9	2.65 (0.67, 17.8)	0.2
Race										
White	—		—		—		—		—	
Other/non-white	-8.6 (-13, -4.0)	<0.001	0.71 (0.45, 1.13)	0.15	0.66 (0.39, 1.09)	0.11	0.75 (0.48, 1.17)	0.2	1.11 (0.47, 2.37)	0.8
Food Security Status										
Food secure	—		—		—		—		—	
Food insecure	-10 (-15, -5.9)	<0.001	0.60 (0.38, 0.94)	0.025	0.67 (0.41, 1.09)	0.11	0.83 (0.53, 1.28)	0.4	0.82 (0.35, 1.78)	0.6

OR = Odds Ratio

1. N=614
2. N=607

Table S2. Stratified Interaction WQSRS. Results from WQSRS analyses stratified and/or testing for interaction on food security status, adjusted for covariates.

Index	Constraint	Stratified	Interaction	Term	Estimate/OR	Std. Error	P-value
MNI[1]	NA	Yes	No	WQS	7.96	3.89	0.04
	Positive	Yes	No	WQS	9.01	4.12	0.03
		No	Yes	WQS	2.25	1.85	0.226
				WQS*Food insecurity	3.1	4.58	0.498
		Yes	Yes	WQS	6.52	4	0.103
				WQS*Food insecurity	2.05	7.35	0.78
Electrolyte index[2]	NA	Yes	No	WQS	1.12	0.421	0.00763
	Positive	Yes	No	WQS	1.12	0.421	0.00754
		No	Yes	WQS	2.49	5.03	1.00
				WQS*Food insecurity	1.00	1.00	1.00
		Yes	Yes	WQS	2.37	1.43	0.0151
				WQS*Food insecurity	1.30	2.42	0.769

1. N=623
2. N=624.

SUPPLEMENTARY FIGURES

Figure S1. Sankey Plot without Nodes. Sankey Plot illustrating the overlap of genera with weights above the threshold (summed OTU weights within genera) from each of the 6 statistically significant WQS_{RSRH} analyses testing for association between the microbiome mixture and the nutritional indices. The genera represented show that each genus was found to be highly weighted. Nodes, from right to left, display the WQS_{RSRH} analysis outcome (My Nutrition Index/Electrolyte Index) with stratification of WQS_{RSRH} weights by food security (not stratified/food secure/food insecure), and bacterial genus.

Figure S2. MNI WQS_{RSRH} OTU Weights. Sum of OTU mean weights within genera across 100 repeated holdouts above threshold (sorted by phylum) for the MNI WQS_{RSRH} analysis. Data points indicate sum of weights within genera for each of the 100 holdouts. Box plots show 25th, 50th, and 75th percentiles of the sum of the weights within genera for the 100 holdouts. Closed diamonds show sum of mean weights within genera for the 100 holdouts.

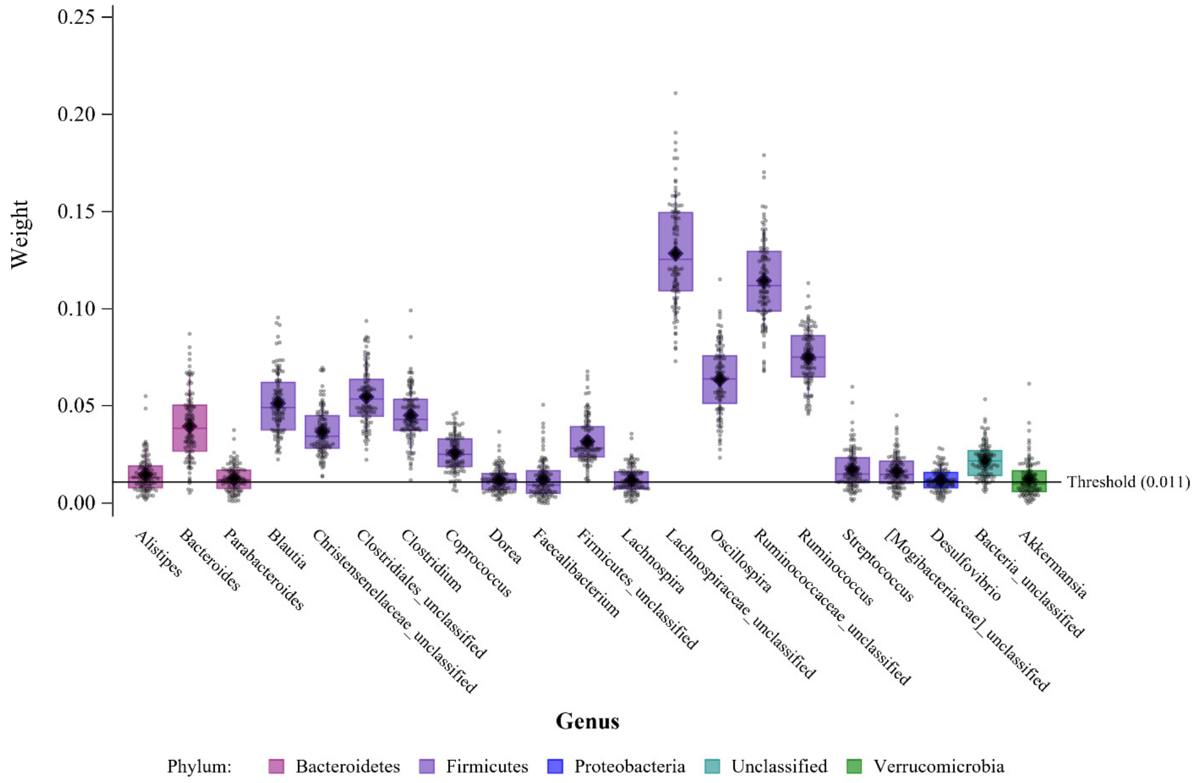

Weight
0.25
0.20
0.15
0.10
0.05
0.00
Threshold (0.011)
Alistipes
Bacteroides
Parabacteroides
Blautia
Christensenellaceae_unclassified
Clostridiales_unclassified
Clostridium
Coprococcus
Dorea
Faecalibacterium
Firmicutes_unclassified
Lachnospira
Lachnospiraceae_unclassified
Oscillospira
Ruminococcaceae_unclassified
Ruminococcus
Streptococcus
[Mogibacteriaceae]_unclassified
Desulfovibrio
Bacteria_unclassified
Akkermansia
Genus
Phylum:
Bacteroidetes
Firmicutes
Proteobacteria
Unclassified
Verrucomicrobia

Figure S3. Electrolyte Index WQS_{RSRH} OTU Weights. Sum of OTU mean weights within genera across 100 repeated holdouts above threshold (sorted by phylum) electrolyte index WQS_{RSRH} analysis. Data points indicate sum of weights within genera for each of the 100 holdouts. Box plots show 25th, 50th, and 75th percentiles of the sum of the weights within genera for the 100 holdouts. Closed diamonds show sum of mean weights within genera for the 100 holdouts.

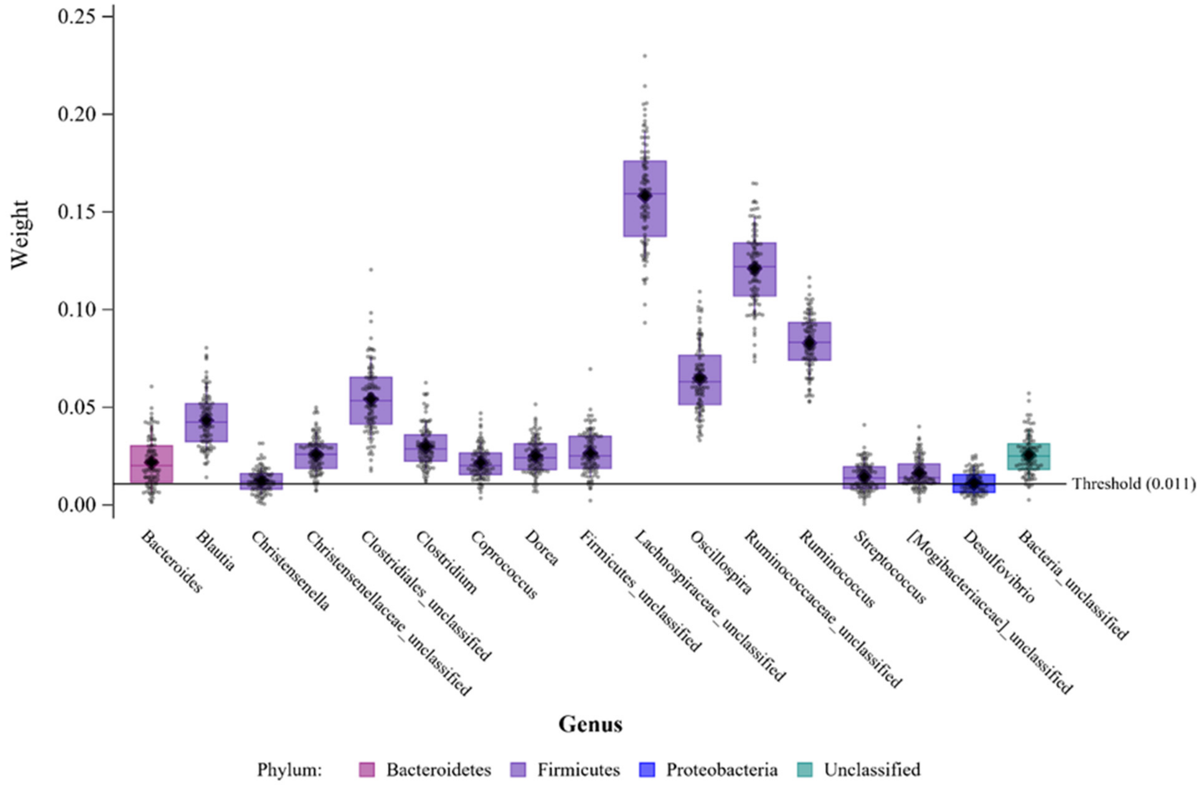

Figure S4. MNI Stratified WQS_{RSRH} Weights. Genera-specific weights from MNI stratified WQS_{RSRH} with weights above the threshold.

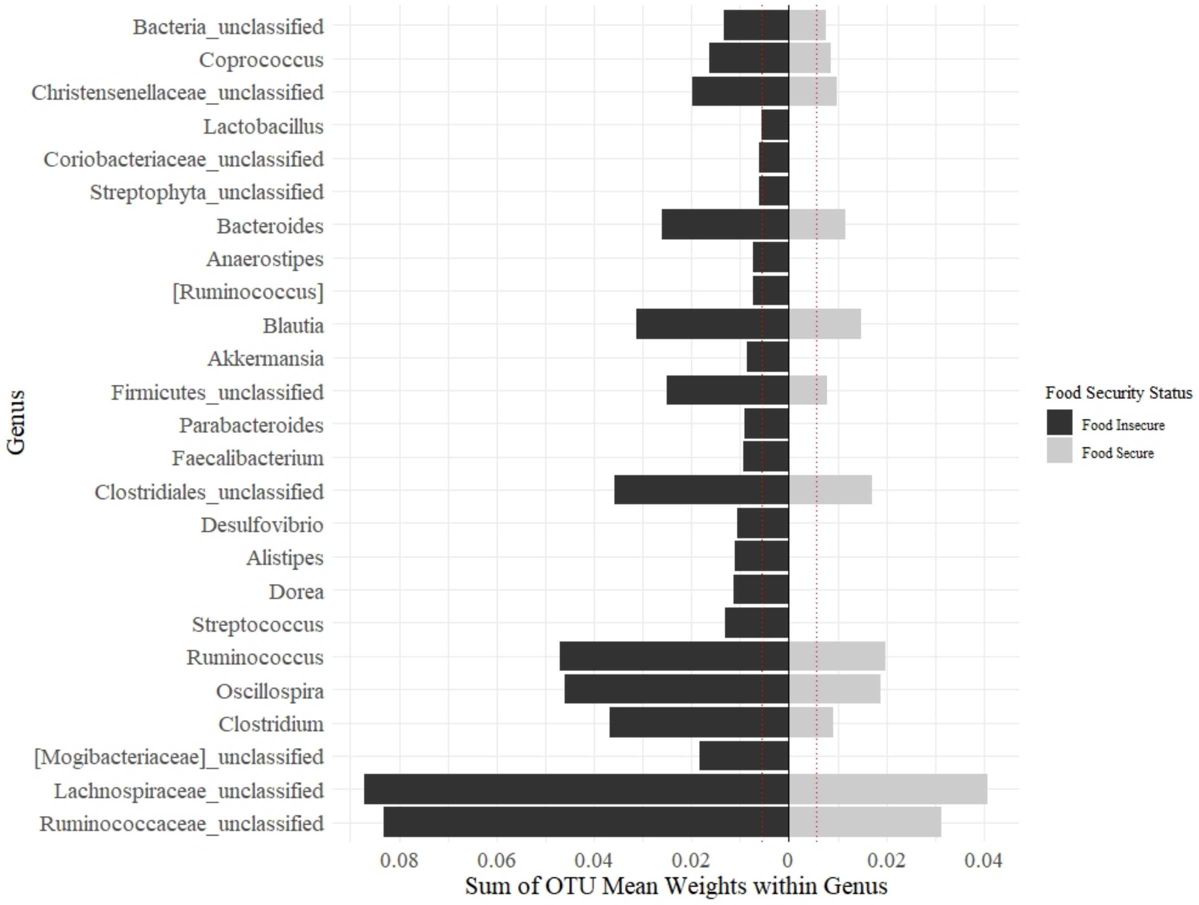

Figure S5. Electrolyte Index Stratified WQS_{RSRH} Weights. Divergent plot from OTU weights summed within genera for the electrolyte index stratified WQS_{RSRH} analysis with weights above threshold

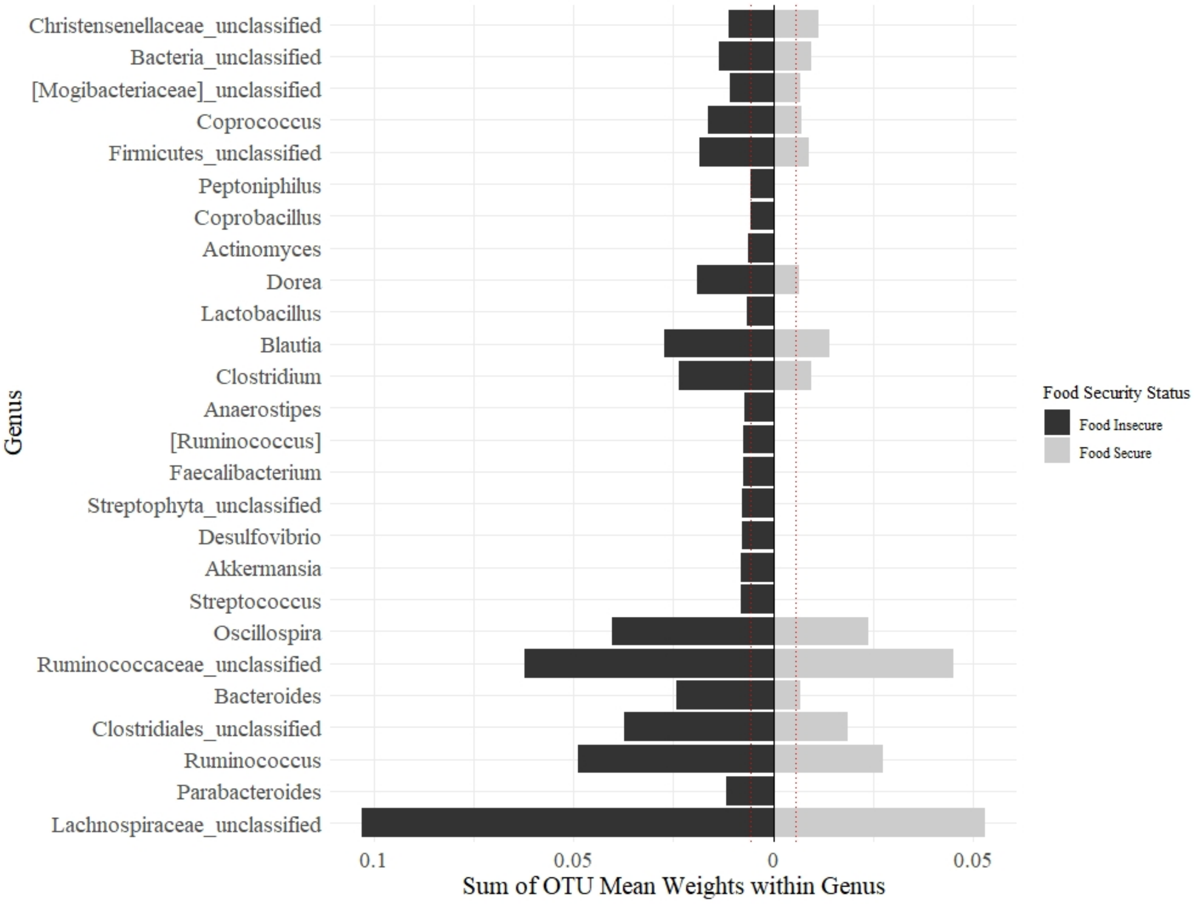

Supplement: Supplementary file 1 [file nutrients-14-03407-s001.zip › Supplemental Tables and Figures.pdf]
