# Supplementary material for: Individual Nutrition Is Associated with Altered Gut Microbiome Composition for Adults with Food Insecurity
Source: Nutrients. 2022 Aug 19;14(16):3407. doi: 10.3390/nu14163407 (PMC9416073; doi:10.3390/nu14163407)
Supplement: Supplementary file 1 [file nutrients-14-03407-s001.zip › Supplemental Text.pdf]

SUPPLEMENTAL METHODS

My Nutrition Index Subscales

There are four MNI subscales. The Electrolyte Index includes sodium, potassium, and chloride. The Macro Nutrient Index includes protein, carbohydrates, and fat. The Mineral Index includes calcium, iron, magnesium, phosphorus, manganese, zinc, and selenium. The Vitamin Index includes Vitamin A, Vitamin B5, Vitamin B6, Vitamin B12, Vitamin C, Vitamin D, Vitamin E, Vitamin K, niacin, riboflavin, and thiamin. Each subscale is scored 0 to 100, the same as the MNI scale, with higher scores indicating better nutrition in that dietary category.

Covariate Selection

Additional covariate selection was performed using generalized linear models with the following covariates in one model versus each calculated index as our outcomes: education level (<high school, high school or associates degree, bachelor's degree or above--< high school is referent group), race (nonwhite vs. white), poverty income ratio, and food insecurity (food insecure vs. food secure). If each variable had a p-value <0.1 when adjusted for all other variables listed, the covariate was maintained in further analyses.

Microbiome Data Processing

The microbiome data was selected from participants with complete covariate and outcome data for further analyses. Any operational taxonomic units (OTUs) with less than 10% of the total taxonomic units detected were excluded from further analyses. Two participants had 2 microbiome samples each. Each OTU was averaged between the two samples. Further analyses

were carried out at the OTU level regardless of the taxonomic accuracy, such that OTUs identified at the species level were included, as were OTUs identified at the phylum level.

Preliminary WQS Models

All preliminary analyses were performed using WQS_{RS}, in which case we did not constrain the estimation for the weights in relation to the outcome to have a positive association or a negative association. Dependent on the results, if the un-constrained results showed a statistically significant association between the microbiome mixture and the given outcome adjusted for covariates, we queried how many times the weight estimations were positively associated with the outcome versus negatively associated. If in ~75% of the results the weights were positively associated with the outcome, we performed further WQS_{RS} analyses with constraints in the positive direction (and vice versa).

When associations were statistically significant two more times using unique random seeds, the WQS_{RS} analyses were extended to the repeated holdout extension. Upon statistical significance in the WQS_{RSRH} analyses, further exploration of the potential confounder and effect modifier were employed such that the WQS_{RS} analyses stratified by food insecurity were assessed. WQS_{RS} analyses with an interaction term for the WQS index by food insecurity were assessed for effect modification by food security status, and a stratified interaction analysis was assessed in WQS_{RS}. Again, upon statistical significance in any of these analyses using the WQS_{RS}, the repeated holdout extension was employed on the same analysis to increase the power and generalizability of the analysis.

SUPPLEMENTAL RESULTS

MNI Preliminary WQS Models

We found a marginally significant positive association between the WQS_{RS} microbiome mixture and the MNI when analyzed with no constraints across 1,000 subsets, in which 82% of the results had positive associations. Constrained in the positive direction, the WQS_{RS} microbiome mixture on the MNI then had a significant positive association after adjustment for covariates ($\beta=3.95$, $p=0.03$), indicating that for each decile increase in the microbiome WQS index, there was approximately a 4-unit increase in the MNI.

Subscale Preliminary WQS Models

Similar to the MNI, we found a significant positive association between the WQS_{RS} microbiome mixture and the electrolyte index after adjustment for covariates (OR=1.62, $p=0.007$), indicating that for each decile increase in the microbiome WQS_{RS} index, there was approximately a 60% increase in the odds of having an above median electrolyte index score versus a below-median score.

The macronutrient, vitamin, and mineral indices had no statistically significant associations with the WQS_{RS} microbiome mixture.
